# PReS-FINAL-2277: Clubbing fingers in a boy with arthritis

**DOI:** 10.1186/1546-0096-11-S2-P267

**Published:** 2013-12-05

**Authors:** T Giani, G Vannucci, M Davide, E Marrani, A Paladini, G Simonini, I Pagnini, R Cimaz

**Affiliations:** 1 Rheumatology Unit, Anna Meyer Children Hospital, Florence, Italy

## Introduction

Hypertrophic osteoarthropathy is a syndrome characterized by clubbing of the fingers and toes, periostosis of long bones, pain and swelling of the joints, and, in more advanced instances, pachydermia. The syndrome is often secondary to cardio-pulmonary or intestinal diseases.The primary form, known as pachydermoperiostosis, is a rare genetic disorder with autosomal dominant (with incomplete penetrance) or recessive transmission, for which there is no specific therapy.

## Objectives

A 10-yr-old Caucasian boy was referred to our department due to swelling of the right knee and the presence of persistent pain for two months associated with morning stiffness.

## Methods

Family history was not contributory. Examination of the joints confirmed arthritis of his right knee. Further physical examination revealed evident clubbing of all his fingers and toes, and a palmo-plantar hyperhydrosis. Examination of his chest and abdomen was unremarkable. All laboratory results including inflammatory markers, complete blood count and other routine biochemistry were within the normal range. Autoantibody screening assays were negative. A high resolution CT scan of the chest, an echocardiogram, and pulmonary function tests showed no pathological results. An x-ray of his femur revealed a mild periostal hypertrophy.

## Results

We decided to treat the patient with non steroidal anti-inflammatory drugs to limit joint inflammation, but the persistence of arthritis led us to perform an intra-articular steroid injection, with good results.

## Conclusion

Because of articular manifestations rheumatologists should be able to distinguish hypertrophic osteoarthropathy from chronic rheumatic diseases and to discern the primary from the secondary form.

## Disclosure of interest

None declared.

